# Endoscopic transcranial transdiaphragmatic approach in a single-stage surgery for giant pituitary adenomas

**DOI:** 10.3389/fonc.2023.1133861

**Published:** 2023-02-01

**Authors:** Xuechao Wu, Zhongyuan Bao, Wei Tian, Jing Wang, Zengli Miao, Qing Wang, Xiaojie Lu

**Affiliations:** ^1^ Department of Neurosurgery, Jiangnan University Medical Center, Wuxi, China; ^2^ Wuxi Neurosurgical Institute, Wuxi, China; ^3^ Department of Neurosurgery, Wuxi No.2 People’s Hospital, Affiliated Wuxi Clinical College of Nantong University, Wuxi, China

**Keywords:** endoscopy, transcranial transdiaphragmatic approach, giant pituitary adenoma, gross total resection, single-stage

## Abstract

**Background:**

The treatment for giant pituitary adenomas (GPAs, maximal diameter >4 cm) remains challenging, with remarkable mortality and morbidity, and there is no consensus on the optimal surgical approach. Gross total resection (GTR) for GPAs is difficult to achieve through a single transsphenoidal or transcranial approach. Any residual tumor is at risk for postoperative apoplexy. In this study, we propose a new surgical technique for resecting the GPAs in a sing-stage transcranial surgery.

**Methods:**

A retrospective review of 4 patients with complicated GPAs, who had been treated *via* an endoscopic transcranial transdiaphragmatic approach in a single-stage surgery after routine transcranial resection, was performed. The following data was analyzed: clinical characteristics, preoperative imaging studies, resection rate, perioperative morbidity and mortality, as well as postoperative outcomes.

**Results:**

All patients had nonfunctioning GPAs and preoperative visual disturbances. In three patients, GTR was achieved, and in one patient, near-total resection (90%-100% of the tumor) was achieved. Three patients attained improved postoperative visual function, while one patient’s vision remained unchanged. One patient suffered a deficiency in adrenocorticotropic hormone along with thyroid-stimulating hormone, and one patient developed diabetes insipidus. Notably, none of the patients suffered cerebrospinal fluid leakage. However, one patient developed an epidural hematoma and underwent decompressive craniectomy.

**Conclusions:**

The endoscopic transcranial transdiaphragmatic approach in a single-stage surgery can be efficiently and safely performed for maximal excision of GPAs with extensive suprasellar extension. Furthermore, relative to the conventional combined or staged approaches, this innovative surgical strategy provides neurosurgeons with a clear operative field with reduced invasiveness.

## Introduction

GPAs (giant pituitary adenomas) are tumors with a maximal diameter of more than 4 cm, accounting for 6%-10% of all pituitary adenomas (1). The most common presentation of GPAs includes visual impairment, headache, endocrine dysfunction, as well as cranial nerve palsy (2). Despite the advancements in modern neurosurgical strategies in the surgical intervention of pituitary adenomas, surgical treatment of GPAs remains a great challenge in terms of gross total resection (GTR) and complication rates because of the large tumor size, irregular shape, suprasellar extension, and cavernous sinuses invasion (3). Peritumoral swelling caused by subsequent bleeding from the residual tumor may lead to deteriorated neurologic and visual outcomes, as well as higher morbidity (4, 5).

Traditionally, the principal surgical approaches for treating GPAs have been microscopic transsphenoidal and various transcranial procedures (6). With the development of the extended endoscopic endonasal approach (EEA) in the previous decade, it is now feasible to achieve greater rates of complete resection, preservation of pituitary function, improved visual outcomes along with lower rates of cranial nerve impairment in individuals with GPAs (5, 7). Therefore, EEA is gradually becoming the first-line treatment for GPAs. However, due to the anatomic restrictions, this approach is difficult to effectively resect GPAs with extensive superior-lateral extension and multilobular shapes (3). Recently, numerous authors have proposed a simultaneous combined transsphenoidal along with transcranial approach through microscopic and/or endoscopic operation to optimize the resection of such complicated GPAs with extensive suprasellar extension (8–11). However, the risks and benefits should be carefully considered before conducting a simultaneous combined approach because it also has disadvantages, including a higher risk of infection, a longer operation time, and possible complications linked to both transsphenoidal and transcranial approaches.

In our experience, the transcranial approach may be more suitable and safer for some selected tumors with irregular expansions into the third ventricle, frontal, or temporal lobes, because open craniotomy allows a larger surgical area. However, conventional microscopic surgery is difficult to remove the intrasellar portion of tumors from the above approach due to the limited visualization under the sella diaphragm, leading to residual tumor and recurrence. Hence, in this study, we present a new surgical technique, which is less invasive and much simpler than traditional combined approaches. In the transcranial surgery for GPAs with excessive suprasellar invasion but a small intrasellar portion, we first resected the suprasellar tumor using a microscope or endoscope. Then, we dissected the sella diaphragm and removed the intrasellar tumor using an endoscope through the craniotomy surgical corridor. Herein, we detail the technical nuances of the endoscopic transcranial transdiaphragmatic approach in a single-stage surgery in managing selected GPAs and describe its applications and limitations. We expect that this approach may improve the opportunities of attaining a GTR with a single surgical procedure.

## Materials and methods

### Design of study

A retrospective study was carried out to review all patients of GPAs with extensive suprasellar extension, who underwent endoscopic transcranial transdiaphragmatic approach in a single-stage surgery at our institution from 2018 to 2020. Patients’ radiological imaging, visual status, and endocrinological evaluation were assessed pre-and postoperatively. This study was approved by the institutional ethics committee, and written consent was provided by all patients.

### Tumor resection by routine transcranial surgery

The frontobasal interhemispheric approach and pterional approach were utilized in this study on the basis of the tumor growth pattern and neurosurgeon’s preference. The side chosen for craniotomy was decided according to the direction of tumor extension and invasiveness. The frontobasal interhemispheric approach was carried out by the coronal skin incision behind the hairline and a paramedian unifrontal craniotomy. The dural flap was subsequently rotated medially from the base, with the major bridge vein to the midline or superior sagittal sinus being protected. For the pterional approach, a standard frontotemporal craniotomy was conducted with the sphenoid wing being drilled. The dura was opened curvilinearly towards the base, and cerebrospinal fluid (CSF) was released by sharp dissection of the sylvian fissure to expose the tumor adequately in the suprasellar area. The frontal lobe was lifted gravitationally with less retraction force with sufficient CSF drainage. Following craniotomy, we carried out tumor resection within the tumor capsule to avoid harming the perforating arterial branches that supply the optic apparatus and hypothalamus through various corridors of each approach, including the interoptic, interhemispheric, optico-carotid, as well as carotico-oculomotor spaces.

### Tumor resection by endoscopic transdiaphragmatic approach

**Figure 1:**
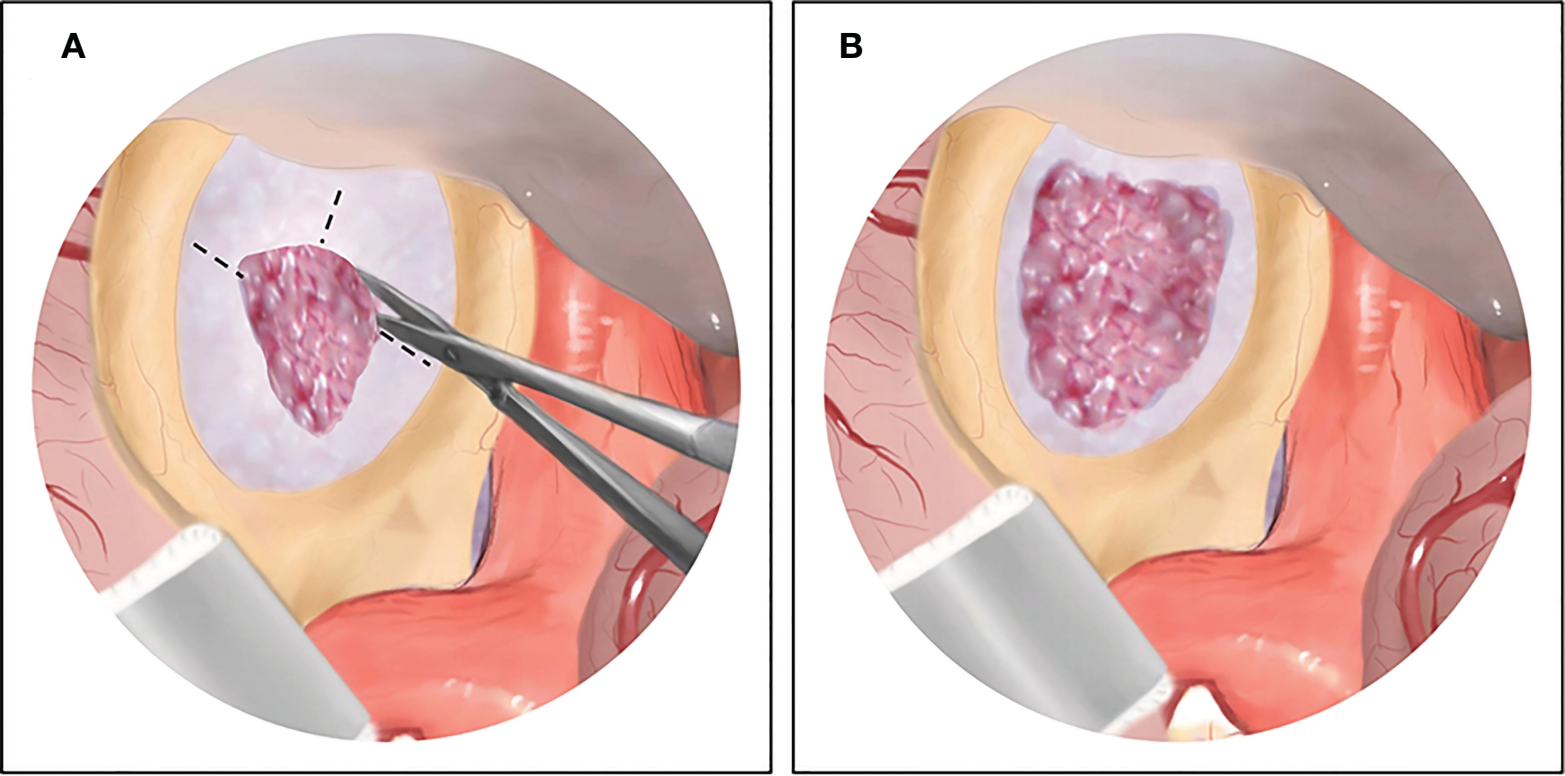
The illustration of surgical maneuvers of endoscopic transcranial transdiaphragmatic approach. **(A)** After complete excision of the suprasellar tumor by conventional transcranial surgery, the dilated diaphragma sellae is exposed, and then dissected anteriorly and laterally. **(B)** The diaphragma sellae is cut open and the residual tumor beneath the diaphragma sellae is clearly visible under endoscopic view.

After the complete excision of the suprasellar component of the tumor under a microscope or an endoscope, the dilated diaphragma sellae was exposed. Then, the diaphragma sellae was cauterized using bipolar coagulation, and dissected along the direction from the center to the outside of diaphragma sellae opening to expand the operative corridor into the sella turcica until the residual tumor beneath the diaphragma sellae was clearly visible. Surgical maneuvers are shown in **Figure 1**. At this point, we introduced a 0° endoscope into the sella fixed with a pneumatic Point Setter (Storz, Tuttlingen, Germany). Under the endoscopic view, the intrasellar tumor was resected in order from the front side to the bilateral sides of the sella turcica by using a combination of curved curettes and suctions. During the resection of the tumor located posteriorly within the sella turcica, a 30° endoscope was placed through the diaphragm sellae opening to help identify and preserve the pituitary stalk. In this way, the intrasellar tumor can be completely removed under a clear endoscopic view in a single-stage transcranial surgery.

## Results

### Patient characteristics

The detailed characteristics of all patients are summarized in **Table 1**. All patients (N=4; 3 men and 1 woman; mean age, 57 years) exhibited visual dysfunction. Two patients suffered from dizziness. Hydrocephalus was present in 1 case. All patients had no prior surgery. All tumors were nonfunctioning pituitary adenomas and exhibited a multilobular shape. Based on the preoperative MRI, the mean maximum diameter was 59.7 mm (range from 55.1 to 65.1 mm). The suprasellar extension was evaluated by assigning Hardy-Wilson (12) and Goel grades (4). According to the Hardy-Wilson classification, we found 3 grade II and stage D tumors, and 1 grade III and stage D tumor. According to the Goel classification, all tumors belonged to grade IV.

**Table 1:** Characteristics of patients.

Case	Age (years)	Sex	Initial Symptoms	Tumor Type	Tumor Configuration	Maximum Diameter (mm)	Hardy-Wilson stage	Goel Grade
1	57	Male	Visual disturbance	NF	Multilobular	55.1	II D	IV
2	67	Male	Visual disturbance, dizziness	NF	Multilobular	65.1	II D	IV
3	40	Female	Visual disturbance, headache, dizziness	NF	Multilobular	62.3	III D	IV
4	64	Male	Visual disturbance	NF	Multilobular	56.2	II D	IV

NF, nonfunctioning adenoma.

### Postoperative results

Postoperative results are summarized in **Table 2**. The rate of tumor resection was categorized as gross total resection (GTR, 100%), near-total resection (NTR, 90%-100%), subtotal resection (70%-90%), and partial resection (<70%) according to Juraschka et al. (5). In this study, GTR was attained in 3 patients, and NTR in 1 patient. The operation duration ranged from 5.75 to 8 hours. Particularly, 3 patients had markedly improved postoperative visual function. The other patient had a stable visual function. The endocrinologic assessment revealed that 1 patient developed persistent diabetes insipidus, while another had a deficiency of thyroid-stimulating hormone along with adrenocorticotropic hormone. Both patients required hormone replacement therapy. A serious complication occurred in 1 patient who suffered an epidural hematoma that underwent decompressive craniectomy by removing the bone flap. During the follow-up period (8 to 63 months), no obvious tumor relapses were evident in all these cases.

**Table 2:** Operative procedures and patient outcomes.

Case	Cranial Approach	tumor resection rate	Operation Time	Postoperative Visual Function	New Hormonal Deficit	Other Complications	Follow-Up (Months)
1	Pterional	GTR	5h 45min	Improved	None	None	43
2	frontobasal interhemispheric	GTR	6h	Improved	None	None	36
3	frontobasal interhemispheric	NTR	6h 30min	Improved	diabetes insipidus	None	20
4	Pterional	GTR	7h 30min	No change	TSH, ACTH	extradural hematoma	10

GTR, gross total resection; NTR, near total resection; TSH, thyroid-stimulating hormone; ACTH, adrenocorticotropic hormone.

### Case illustration

#### Case 1

**Figure 2:**
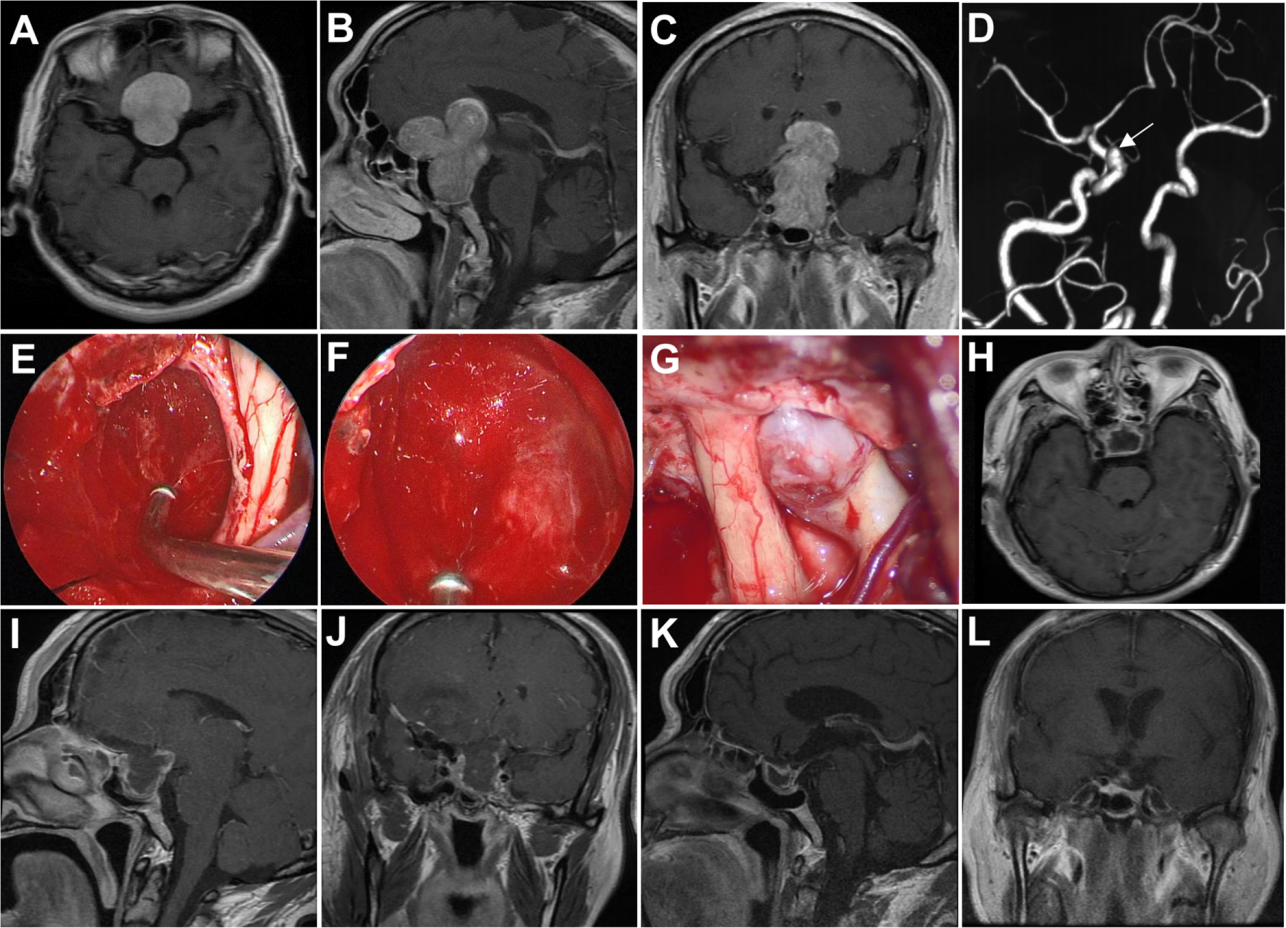
Case 1. Pre- and postoperative imaging findings and intraoperative images. **(A–C)** Preoperative axial **(A)**, sagittal **(B)** and coronal **(C)** gadolinium-enhanced T1-weighted MRI images showing a giant pituitary adenoma with extensive suprasellar extension. **(D)** Preoperative CTA examination reveals an aneurysm located in the C6 segment of the right internal carotid artery. The white arrow indicates the aneurysm. **(E)** Intraoperative photograph showing that the tumor in the intrasellar part is removed with angled suctions under endoscopic view. **(F)** Intraoperative endoscopic photograph showing the final view after removal of the intrasellar tumor. **(G)** Intraoperative microscopic photograph showing the aneurysm. **(H–J)** Postoperative axial **(H)**, sagittal **(I)** and coronal **(J)** gadolinium-enhanced T1-weighted MRI images showing gross total resection of the pituitary tumor two days after surgery. **(K, L)** Postoperative sagittal **(K)** and coronal **(L)** gadolinium-enhanced T1-weighted MRI images obtained 4 months after surgery.

A 57-year-old man presented with progressive worsening visual acuity in both eyes of 8 years’ duration and loss of temporal vision in the left eye in the last year. MRI revealed a giant sellar and suprasellar mass with a maximum diameter of 55.1 mm and extensive extension into the frontal lobe. Additionally, a preoperative CTA examination revealed an aneurysm of 4 mm located in the C6 segment of the internal carotid artery. A traditional pterional approach was performed for the tumor resection and aneurysm clipping. After the suprasellar tumor was removed by using the microscope, we enlarged the surgical corridor by dissecting the sellar diaphragm to remove the residual intrasellar tumor under a 30°endoscope. Lastly, the aneurysm was clipped under the microscopic view. Gross total resection was achieved, and postoperative visual acuity improved markedly. MRI at 4 months following surgery showed no recurrence of the tumor (**Figure 2**).

#### Case 2

**Figure 3:**
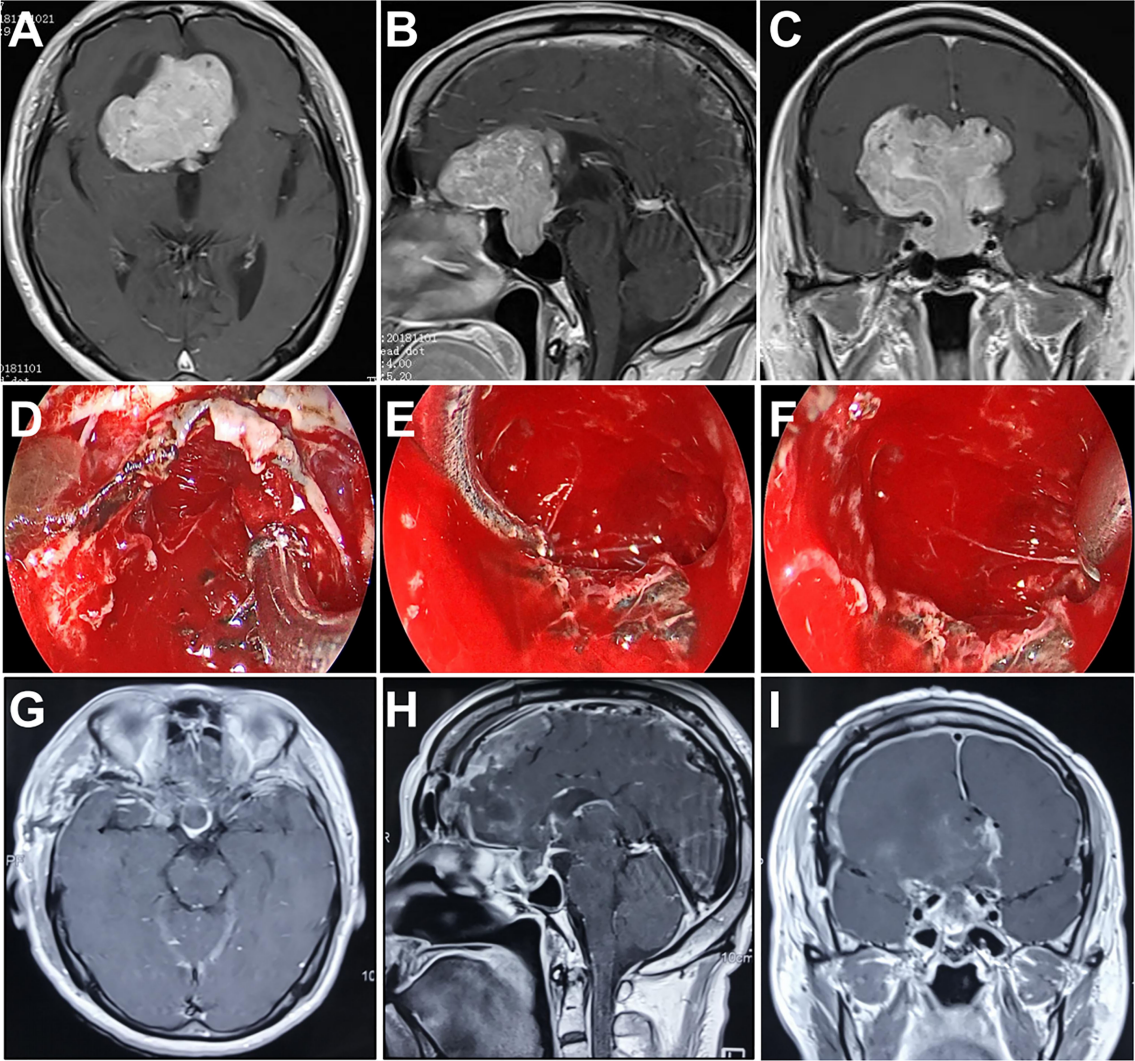
Case 2. Pre- and postoperative imaging findings and intraoperative images. **(A–C)** Preoperative axial **(A)**, sagittal **(B)** and coronal **(C)** gadolinium-enhanced T1-weighted MRI images showing a giant pituitary adenoma with extensive suprasellar extension. **(D)** Intraoperative photograph showing that the diaphragma sellae is sharply opened and the intrasellar tumor is removed with angled suctions under endoscopic view. **(E, F)** Intraoperative endoscopic photographs showing the final view of the medial walls of both sides of cavernous sinus as well as the anterior wall of sella turcica after removal of the tumor. **(G–I)** Postoperative axial **(G)**, sagittal **(H)** and coronal **(I)** gadolinium-enhanced T1-weighted MRI images showing gross total resection of the pituitary tumor.

A 67-year-old man presented with dizziness, nausea, and blurred vision over 2 months. MRI revealed a giant tumor with extensive expansion leading to severe mass effect. The maximum diameter of tumor was 65.1 mm. An endocrine workup showed that he had low adrenocorticotropic hormone (ACTH). We selected the frontobasal interhemispheric approach due to the invasion with multiple lobes into the subarachnoid space, as well as the extension of the tumor anteriorly over the planum sphenoidale. A complete resection was obtained by using the endoscopic transcranial transdiaphragmatic approach without any severe complications. He had improvement in his vision and ACTH levels postoperatively (**Figure 3**).

#### Case 3

**Figure 4:**
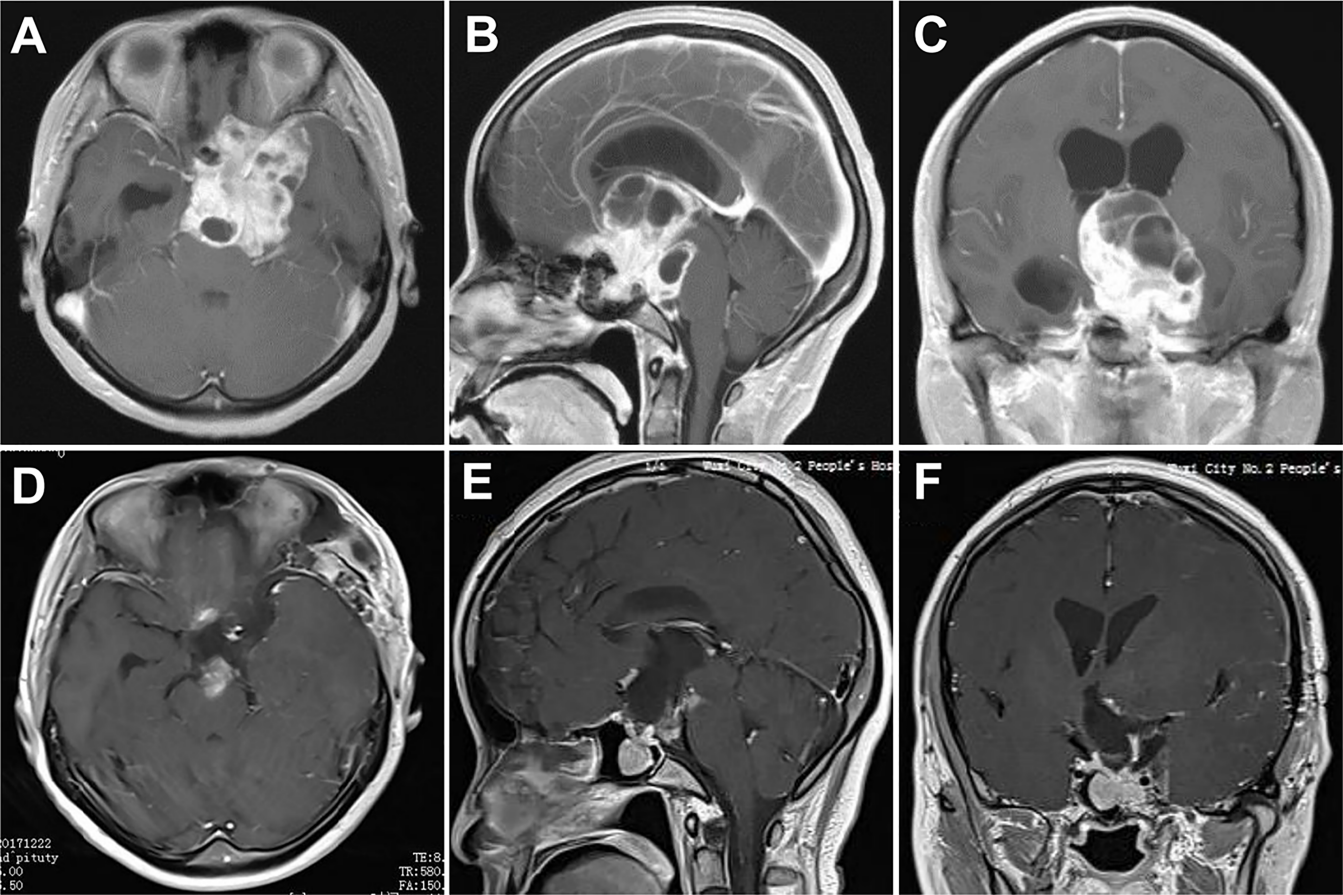
Case 3. Pre- and postoperative imaging findings. **(A–C)** Preoperative axial **(A)**, sagittal **(B)** and coronal **(C)** gadolinium-enhanced T1-weighted MRI images showing a giant pituitary adenoma with extensive suprasellar extension. The tumor fills the entire third ventricle and invades into the sphenoid sinus. **(D–F)** Postoperative axial **(D)**, sagittal **(E)** and coronal **(F)** gadolinium-enhanced T1-weighted MRI images showing near total resection of the pituitary tumor with residue in the sphenoid sinus.

A 40-year-old woman presented with severe headache and dizziness, as well as progressive visual deterioration over 3 weeks. MRI analysis exhibited a giant sellar and suprasellar mass with multicystic components. The tumor extended directly to the third ventricle and caused hydrocephalus. Besides, the tumor focally perforated the floor of the sella and invaded into the sphenoid sinus. We decided to adopt the endoscopic transcranial transdiaphragmatic approach in a frontobasal interhemispheric procedure for tumor resection. The surgery was uncomplicated, and NTR was achieved with residual tumor hidden inside the sphenoid sinus (**Figure 4**). The patient’s hydrocephalus improved after the operation but was complicated by diabetes insipidus requiring long-term replacement therapy.

#### Case 4

**Figure 5:**
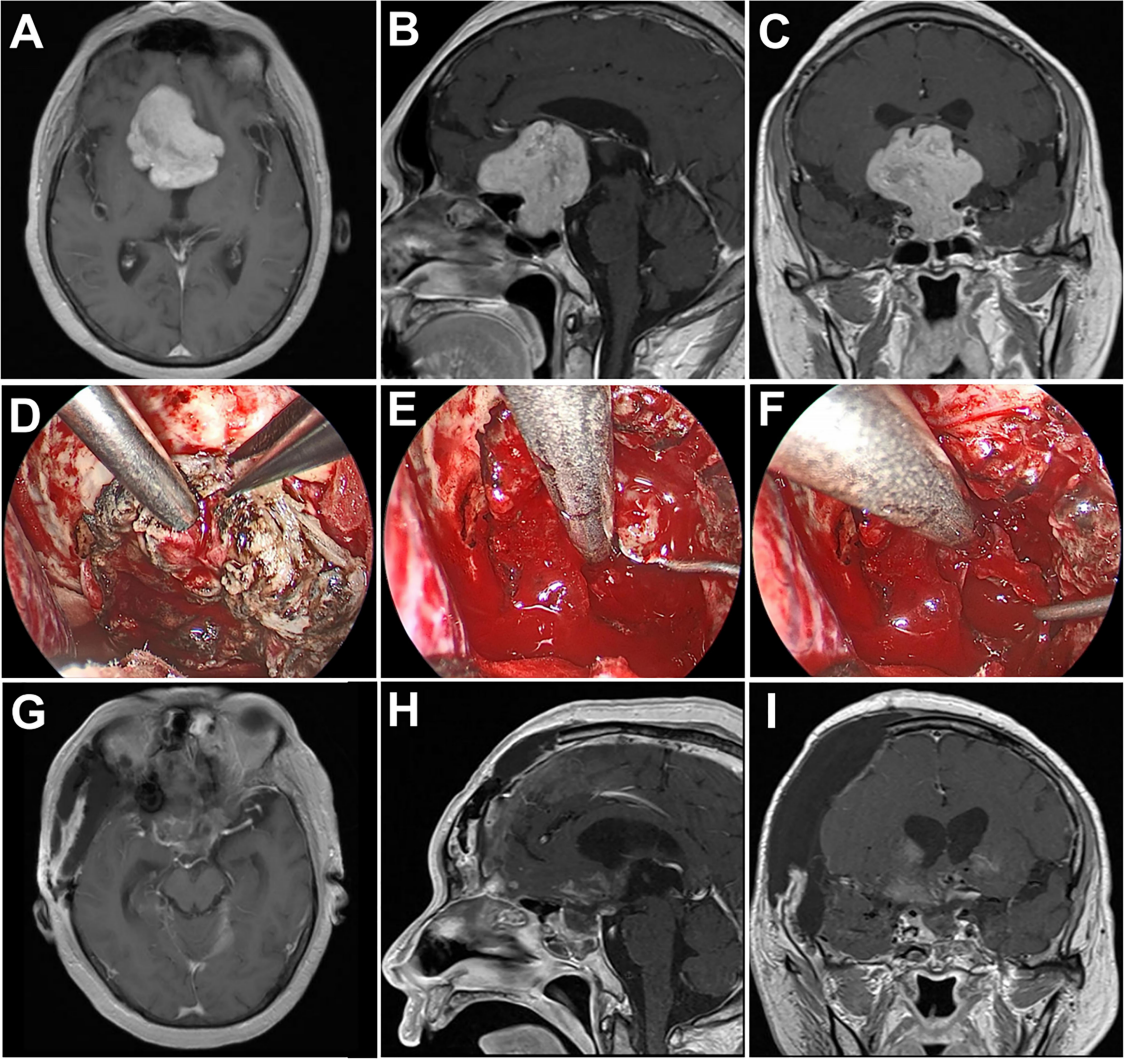
Case 4. Pre- and postoperative imaging findings and intraoperative images. **(A–C)** Preoperative axial **(A)**, sagittal **(B)** and coronal **(C)** gadolinium-enhanced T1-weighted MRI images showing a giant pituitary adenoma with extensive suprasellar extension. **(D)** Intraoperative photograph showing that the diaphragma sellae is cut with micro-scissors. **(E, F)** Intraoperative endoscopic photographs showing the final view of the medial walls of both sides of cavernous sinus as well as the anterior wall of sella turcica after removal of the tumor. **(G–I)** Postoperative axial **(G)**, sagittal **(H)** and coronal **(I)** gadolinium-enhanced T1-weighted MRI images showing gross total resection of the pituitary tumor.

A 64-year-old man presented with progressive visual deterioration in both eyes for 3 years. Preoperative MRI revealed a giant tumor with considerable suprasellar extension compressing the anterior cerebral arteries along with the optic apparatus. A fully endoscopic pterional approach was performed and the tumor was completely resected. His vision in both eyes improved significantly after surgery. However, this patient developed progressive epidural hematoma postoperatively and presented a coma 7 days after the operation. An emergent decompressive craniectomy was performed to evacuate the hematoma, and remove the original bone flap due to brain swelling. The patient recovered well from surgery with mild hypopituitarism (**Figure 5**).

## Discussion

### Routine transcranial surgery for GPAs

The treatment goal of GPAs is mainly complete resection, partial resection plus adjuvant radiotherapy, or a combination of surgery, and radiotherapy along with observation. Surgical resection of GPAs is still very challenging because of their enormous size, multilobular shape, and irregular extension into the cavernous sinuses or suprasellar area (1, 13, 14). Each extension has its anatomical characteristics that should be discussed separately. In particular, lateral extensions greatly make surgical access and resection difficult. The postoperative morbidity and mortality of GPAs are mainly caused by damage to perforating arteries or subsequent bleeding from the residual tumor. To prevent postoperative apoplexy, radical resection of the tumor, including GTR and NTR, should be the primary surgical goal. Thus, it is critical to determine the optimal surgical approach to maximally resect GPAs.

Both transsphenoidal and transcranial approaches have been conventionally employed in the surgical treatment of GPAs for many years. Because the suprasellar portion of tumors often descends into the field of operation by the force of gravity following decompression of the intrasellar tumor, the transsphenoidal approach is usually preferred for the majority of GPAs over the transcranial approach (4, 15). During the last decades, the endoscopic endonasal approach enabling removal of GPAs has been increasingly explored, because it offers a detailed, panoramic visualization and wider access to the operative field through the angled endoscopes (3, 16–18). In addition, supplemental imaging using intraoperative MRI, which may detect unanticipated residual tumors and identify the tumor margins in real time, may also improve the rate of GTR during the same surgery (19). Furthermore, the extended endonasal endoscopic approach, which enables removing the bone of tuberculum sella along with a portion of the planum sphenoidale, coupled with exposing the ventral and medial cavernous sinuses, has remarkably improved the total resection rate of GPAs and postoperative visual function (7). However, tumor excision *via* this single approach may be challenging when the tumor extends outside the surgical corridor. Previous literature also suggested that this approach may have limited applications in the treatment of multilobular GPAs because of the absence of extensive linkages between the tumor components (3). Aggressive tumor resection under inadequate visualization may cause injury to adjacent critical structures, as well as lead to severe surgical complications and poor patient outcomes. In addition, postoperative cerebrospinal fluid (CSF) leakage is the most frequent complication of the extended endoscopic endonasal approach. Although the rate of CSF leakage was reported to be reduced to 7.4% after a vascularized nasoseptal flap was routinely used (3), the technical point linked to the difficult multilayer reconstruction of the skull base defect has always rendered the extended endoscopic endonasal approach as a relative challenge.

Considering this complicated situation, various surgical procedures have been reported to optimize resection of GPAs with significant suprasellar and anterior extension, including simultaneous combined surgeries and two-staged surgeries (14). In reality, various types of combinations have been documented: microscopic transsphenoidal approach–microscopic transcranial approach (8), endoscopic transsphenoidal approach–microscopic transcranial approach (10, 20, 21), endoscopic transsphenoidal approach–endoscopic transventricular approach (22), microscopic transsphenoidal approach–endoscopic transventricular approach (23), endoscopic transsphenoidal approach–microscopic transventricular approach (24), and endoscopic transsphenoidal approach– endoscopic supraorbital keyhole approach (11). The greatest advantages of these simultaneous combined surgeries are not only to increase the degree of tumor excision, but also to support safe tumor removal and minimize postoperative bleeding of the residual tumor. However, the potential disadvantages of these simultaneous approaches included the increased risk of infection, invasiveness, and extra craniotomy-linked complications. Moreover, these procedures require twice as many neurosurgeons and twice as many instruments, for instance, microscope and endoscope, which are not available at most institutions.

Some surgeons prefer a 2-staged surgery for difficult GPAs (6, 14, 25), including the utilization of the transsphenoidal approach at first and subsequently the transcranial approach after several months, or vice versa. The prospects of a radical excision are remarkably diminished when more than half of the suprasellar region of the tumor is out of the line of transsphenoidal vision, especially when the tumor consistency is unfavorable (17). Large Hardy’s stage C tumors are a nonhomogenous group of tumors, and several authors have advocated for a staged surgery in these difficult cases to enable the suprasellar residual to descend into the sella (17). The 2-staged surgery procedure also provides a higher rate of tumor excision relative to the single-stage approach. However, the patient will have to undergo surgery twice, and the incidences of postoperative bleeding from the intentionally remaining tumor cannot be avoided.

Although most pituitary adenomas as well as some GPAs can be treated *via* a transsphenoidal procedure, parasellar tumors that are fibrous and adhere firmly to critical structures or that extend far laterally further than the internal carotid artery are still very difficult to remove completely with a transsphenoidal procedure alone. Even though transcranial surgery is usually associated with an elevated risk of postoperative pituitary dysfunction, morbidity and mortality (26), this might be confounded since the transcranial approach is often preferentially selected for more complicated tumors with extensive lateral or anterior extension. The transcranial approach has its own advantages, which are quite useful to reach the lateral extent of tumors, and decompress the neurovascular structures, especially the optic apparatus (26). In fact, the transcranial approach still plays an essential role in about 1-10% of GPAs, and neurosurgeons who deal with numerous cases of pituitary adenomas annually may need to utilize this procedure fairly frequently (25). Shen et al. concluded ten indications for transcranial approaches according to the literature (27), which are 1) tumor mainly located in the suprasellar region and/or the sella turcica was too small and narrow; 2) dumbbell-shaped or hourglass-shaped tumors with a constriction at the level of diaphragma sellae opening; 3) suspicious fibrous consistency; 4) irregular shape with anterior, middle, posterior cranial fossa, or intraventricular extension; 5) brain invasion with cerebral edema; 6) the presence of ectatic carotid arteries projecting toward the midline or coexistent aneurysm; 7) encasement of subarachnoid arteries; 8) postoperative apoplexy in suprasellar residue following transsphenoidal operation; 9) current sinusitis making a transsphenoidal approach inappropriate or previous transsphenoidal surgery; and 10) uncertainty regarding the diagnosis. In our study, all patients had more than 4 of above characteristics. Standardized craniotomies, such as fronto-lateral, pterional and basal midline craniotomies that are close to the skull base, are performed depending on the tumor’s localization to avoid brain retraction. The carotid artery and its branches, as well as the optic nerves and chiasm, may be microscopically dissected with sufficient space created by the drainage of CSF. The transcranial approaches enable the intracranial component of a pituitary tumor to be resected with relative radicality. The major challenge, however, is the risk of damaging the surrounding structures that must be carefully exposed and dissected. In addition, it is difficult to clearly judge whether there is residual tumor in the sella turcica under the narrow microscopic view.

### Advantages of the endoscopic transcranial transdiaphragmatic approach

In this study, we propose a new surgical technique, which is to dissect the sella diaphragm and resect the intrasellar tumor by endoscopic transcranial transdiaphragmatic approach after removal of the suprasellar portion of GPAs. This strategy may help to identify residual tumor within the sella, which can improve the tumor total resection rate, minimize brain retraction, and reduce the possibility of residual tumor apoplexy. In our cohort, we achieved gross total resection in 3 patients, and near total resection in 1 patient. None of the patients had postoperative apoplexy. In addition, this technique eliminates the need for a combined or secondary operation, which is helpful to reduce surgical trauma. Particularly, compared with the endoscopic endonasal approach, the endoscopic transcranial transdiaphragmatic strategy may avoid complicated skull base reconstruction and reduce the risk of CSF leakage and secondary intracranial infection.

### Limitations of the endoscopic transcranial transdiaphragmatic approach

Our study’s limitations include its retrospective aspect and the fact that the cohort was relatively small to make definitive conclusions. Further studies involving more case series are necessary and important to confirm the safety and efficacy of the current approach. This approach needs to overcome all the challenges of resecting GPAs with multiple lobules, extensive invasion, and vascular encasement. This approach is inappropriate for GPAs with a shorter sella turcica length (distance from tuberculum sella to the tip of the dorsum sella). Besides, simultaneous mastering the manipulation of both the microscope and the endoscope requires extensive training and a long learning curve, because dissection of the giant tumors under an endoscopic view remains a great challenge for most neurosurgeons.

## Conclusion

The endoscopic transcranial transdiaphragmatic strategy in a single-stage surgery is an efficacious and safe surgical procedure for a select group of giant pituitary adenomas with extensive suprasellar extension. It is a novel minimally invasive procedure with outstanding postoperative outcomes and a gross total resection rate equivalent to the conventional combined or staged approaches.

## Data availability statement

The original contributions presented in the study are included in the article/supplementary material. Further inquiries can be directed to the corresponding author.

## Ethics statement

Written informed consent was obtained from the individual(s) for the publication of any potentially identifiable images or data included in this article.

## Author contributions

XW and ZB wrote the manuscript. WT and JW analyzed data. ZM, QW, and XL provided data and revised the manuscript. All authors contributed to the article and approved the submitted version.
